# Barriers: The discursive problematization and transformation of HIV-prevention service provision in England (2016-2020)

**DOI:** 10.1057/s41285-025-00246-5

**Published:** 2025-12-11

**Authors:** Adam Christianson

**Affiliations:** 1 Sociology, Goldsmiths, University of London, London, UK; 2 Family Medicine, Faculty of Medicine, University of Manitoba, Winnipeg, Manitoba

**Keywords:** Barriers, Pre-exposure Prophylaxis, England, HIV-prevention, Critical Health, Governmentality

## Abstract

While the statement “patients face barriers” is prevalent in healthcare-related literature, barrier is seldom conceptualized. While reviews of the concept suggest the term is conceptually and methodologically weak, its utility is more nuanced. This article contributes to an emerging critical health literature conceptualizing *barriers* by outlining their discursive effects in healthcare discourses on HIV-PrEP in England between 2017 and 2020. A Situational Analysis of the alliances and positions taken by English stakeholders in debates over PrEP illustrates how designating something as a *barrier* facilitates a power struggle about how healthcare is provided and used. In addition to imparting significance on a problem to patient’s uptake and use of a healthcare intervention, *barriers* align the patient’s problems with groups of experts. Once integrated into an expert discourse, *barriers* facilitate the transformation of healthcare services by objectifying the values, preferences and abilities of a patient community and aligning them with their shared political and governmental aims. *Barriers*, therefore, play an indispensable and strategic role in evidence-based activism and in the broader domains of biological and sexual citizenship with implications for policy and systems management.

## Introduction

*Barriers* are increasingly a feature of healthcare discourse. The term, though consistently deployed to reference a dizzying array of problems is rarely conceptualized. Consequently, the theoretical value of the concept has been questioned (Bach-Mortensen and Verboom, 2020, Haynes and Loblay, 2024). This article responds to this emerging critical literature by examine how barriers operate in healthcare discourse as elements that enable coordination over complex and diverse parts of the healthcare system by designating how and by whom that system is run. Rather than suggest the under-conceptualisation of barriers is a limitation, this ambiguity is strategic. This article draws on a governmentality approach to illustrate how barriers operate as a way of *problematizing* health provision through the needs and values of a patient population, interpreted by representatives and experts. Using a Situational Analysis of the term’s usage in academic and grey literature on biomedical HIV-prevention in England, I illustrate how barriers act as an *empty signifier* that facilitates negotiations over how best to manage healthcare. Authorities capitalize on the ambiguity of barriers to make the interventions malleable for social, political or technological intervention, without engaging in explicit politics and by giving this political debate the semblance of being matters of fact.

The notion that barriers do not simply describe the problems people face accessing care emerges from my own challenges understanding when not accessing or discontinuing HIV-preexposure prophylaxis (PrEP) ought to take place. PrEP is a biomedical intervention that allows HIV-negative persons to prevent HIV acquisition by taking a combination of antiretroviral medications prior to HIV-exposure. Though PrEP has been celebrated for its outstanding efficacy, uptake of the drug in target communities has been slow globally and has been controversial in specialist communities and patient communities alike. These ambivalences have been described in terms of barriers candidates face. Naturally, many people will have reasonable justifications for not using PrEP. Yet, I found that all reasonable explanations concerning the usage of PrEP tended to be indexed by a barrier, making the difference between non-user and candidate ambiguous.

This article examines the determination of the PrEP candidate and the ways in which many of these ambiguities were resolved without appearing explicitly political. By describing the barriers the PrEP candidate faces and those they do not, experts, activists and authorities in HIV care fundamentally transformed healthcare and HIV service provision in England. Their work made publicly administered PrEP based on evidence only, the most desirable option for the British public in general.

## The Case Study: The English PrEP Controversy

I have argued elsewhere that the English health system was organised in such a way to make providing PrEP to the public functionally impossible (Christianson, 2025). The English health system provides low-cost health services to all residents though a national insurance scheme commonly called ‘the NHS’. At the time PrEP was under consideration, ‘the NHS’ in England was fractured into a loose assemblage of discreet and at times competing *commissioning boards*. Reforms undertaken under the coalition government in the 2010s inscribed what I have called an *epidemiological style of thought* in HIV services. Under Health Secretary Lansley, HIV services were split between commissioning boards. HIV positive persons were administered by one board and HIV negative persons by another. Preventative services were also focused on epidemiological risk categories: Men who have sex with men (MSM) and transgender women, drug users, persons who have immigrated from an HIV-endemic country, and persons in serodiscordant relationships. Straddling prevention and treatment, PrEP complicated this arrangement.

These complications created problems almost immediately. Initially, the NHS assigned PrEP to local commissioners, but critics argued this would strain local budgets and create geographic disparities in access. In response, the NHS reviewed their mandate resulted in a subsequent reversal. The decision not to provide PrEP led the National Aids Trust to sue the NHS. The Courts found that the NHS was mandated to provide PrEP. However, the Courts did not provide the NHS with any direction about its administration and its new subjects. To resolve these problems, the NHS sponsored an implementation trial that ran from late 2016 until 2020. The trial delayed routine commissioning, which drew intense criticism from those in the HIV sector for nearly three years.

Conventionally, the story of PrEP in England is told as a victory for the people against the UK Conservative Party’s moralizing and spending cuts (see, Mowlabocus, 2020; Young et al. 2021). More specifically, it exemplifies a victory for United4PrEP, a “collaborative group with wide representation from third sector organizations, clinicians and individual advocates” (Portman, 2016) versus the NHS and Secretary of State for Health. While the state initially withheld an evidence-based, HIV-prevention method from the English public, these collaborative efforts led to its near universal provision through the state healthcare system.

This article deals with another, far less polarized, aspect of this period. Following the judicial review, the NHS prolonged funding the service for three years while they conducted an implementation trial. Some have rightly characterized the period between 2017 and 2020 as a period of heel-dragging by the NHS and Health Ministries in anticipation of PrEP’s patent expiry (Dodds, 2021; Nagginton and Sandset, 2020). But this was not a period of stagnation; activists, health providers and policy makers deliberated hotly in anticipation of PrEP’s commissioning in 2020.

While service funding stalled, people were accessing PrEP outside the NHS in a practice that has been dubbed Do-it-yourself (*DIY) PrEP* (Paperini et al., 2018). The English health system allows individuals to access prescribed drugs privately (MHRA, 2021). Access was facilitated by PrEP-activist websites such as iwantprepnow.com and PrEPster that provided individuals with information on how to use PrEP, how to discuss it with their providers and hosted links to vetted international suppliers. Discourse on PrEP provision also proliferated. Because service funding and drug approvals are independent processes in the UK, physicians’ associations and healthcare oversight groups deemed it necessary to produce local guidance on how and when to prescribe PrEP. Organizations such as the British HIV and Sexual Health Associations (BHIVA/BASHH) and the National Institutes of Healthcare Excellence (NICE) began developing clinical guidance on PrEP. Research on PrEP users, either partaking in clinical trials or accessing PrEP on their own, also proceeded during this time. These debates focused on eliminating or reducing barriers to access and persistence with PrEP through policy, coaching or structural changes to HIV services. Though *barriers* arose multiple times in publications published during this period, it was never conceptualized. Barriers were discussed as if its qualities were so self-evident as to not require definition, let alone a broader theoretical model or approach. But this appearance is deceptive, as the term gives way to concepts and sub-concepts so diverse one might ask—what is not a barrier?

## What are barriers?

Barriers can be structural, interpersonal or individual, and can be perceived, enacted or anticipated. They can reflect a personally held belief or value. Globally, systematic reviews of barriers to PrEP uptake and adherence cite an array of barriers, but the most common are: education, motivation, accessibility, risk-perception, and social stigma (Ching et al., 2020, Coukan et al., 2023, Edeza et al., 2021, Matacotta et al., 2020, Mayer et al., 2020, Pinto et al., 2018). Each of these concepts are themselves indexes for their own array of concepts, that are non-isomorphic. In this respect, barriers can be anything, which makes the concept incredibly flexible, but theoretically and methodologically thin. The emerging opinion on barriers is that this flexibility and tendency to evade definition is a weakness. However, this article makes the case that this mutability and thinness is the strength that allows barriers to function effectively as a discursive object.

Existing theoretical discussion of barriers has found the concept hard to define or model. While some defend the concept as useful, most contend that what counts as a barrier, how and when a barrier interacts with a system depends on the researcher’s perspective. Notable about the literature is a sustained sense that the concept is popular despite its lack of conceptualization, and the protracted deadlock about its definition.

There have been multiple attempts to define, categories and refine barriers. While there were some early attempts at conceptualization in the 1970s (Cummings et al., 1980, Becker, 1976, Haynes et al., 1979), unifying these concepts proved elusive, and these researchers abandoned these efforts by the early 1990s (Daly, 2005). The exponential rise of the *Barriers and Facilitators* approach in the 2010s and 2020s has led to some renewed interest in the concept (Bach-Mortensen and Verboom, 2020, Haynes and Loblay, 2024). However, these studies also note limited progress has been made in definitions. Across studies of barriers in health-systems research Bach-Mortensen and Verboom (2020) have illustrated that the concepts indexed often overlap; the relationship between these concepts are inconsistently fleshed out; they are not generalizable and often change over time—a point echoed in multiple reviews (McCullock Melnyk, 1988, Haynes and Loblay, 2024).

Outside healthcare, similar criticisms of the term have been lodged. Demsetz (1982) argued “barriers to entry” is equally glossed over in economic theory. Barriers are the ‘noise’ that is not explained by the inputs and outputs of a system, but which negatively impacts the system. Economists have characterized barriers as modifiable impediments to individual or social change for a target population in a target social, material and geographic context. In principle modifiable, barriers may be valued differently by the different actors, making them durable across context (Eisenack et al., 2014; Demsetz, 1982). Not only is this definition circular, but it also tells us less about what a barrier is because the same barrier can be interpreted differently in a different context. Likewise, in their debate with Williamson and Nelson (2018), Wellstead et al. (2018) argue that this definition offers little clarity, ie barriers are any object determined to be an impediment to system utilization, wherein impediment is interchangeable with barrier.

Another critique is that barriers are inconsistently theorised. While early conceptualizations of barriers in the healthcare literature were clearly informed by Structural-Functionalism (Becker, 1974, Mykhalovskiy et al., 2004), barriers are more often employed without conceptualization than employed as part of a structural model (McCullock Melnyk, 1988, Haynes and Loblay, 2024). In this regard, Williamson and Nelson (2018) argue barriers are not meant to describe how a system functions but are only empirical descriptions of objective components of complex and dynamic systems, rather than a stable object with specific functions. Health implementation research tends to adopt this empiricist approach, and continues to see the lack of conceptualization as an ongoing limitation of the approach (Bach-Mortensen and Verboom 2020; Haynes and Loblay, 2024).

By contrast, Checkland et al. (2007) argue that barriers serve to justify access to resources and shore up clinical power, rather than objectively describe the problems experienced by healthcare actors. Instead of describing problems, their interviewees used barriers to construct a shared world concerning agreed-upon problems. The metaphor of barriers allows people to understand their social context as well as construct and present a morally acceptable identity. From this vantage point barriers are not simply *anything* that impedes action, nor are they objective descriptions of problematic components of complex systems. Rather, barriers are imbricated in a political discourse aiming to make directed changes. While the Social Constructivist approach strengthens the theoretical underpinnings of the barriers approach, Checkland et al. (2007) do not consider its macro-sociological implications beyond the maintenance of clinical power. Barriers can be critical of clinical power, as exemplified in the literature on barriers to PrEP utilization, wherein the judgements and position of clinicians are heavily criticised (Witzel et al., 2019, Calabrese, 2020, Golub and Fikslin, 2022). Hence, if barriers are political, they are not in service of so-called elites. Though Checkland et al. (2007) offer a more robust explanation for what barriers do, they nonetheless underestimate their value to those who employ them. Barriers are invaluable beyond justifying clinical interests as evidenced by their ubiquitous occurrence in the literature.

## Barriers as an Empty Signifier

I argue that barriers act as a translatory element that enables the coordination between multiple authorities. Nikolas Rose and Peter Miller (1992) have argued that advanced liberal governance is performed “at a distance”, by collecting and disseminating knowledge about the needs, abilities and desires of a population. This knowledge is used to entice the population into willingly complying with governmental priorities, by making prudent, responsible individual choices that allow them to attain their desires. Power is exercised by determining the terms and means by which the individuals desired ends can be attained. Rose and Miller (1992) call this process *problematization*: the process by which governance is achieved through the articulation of the problems and solutions posed by the authorities on a given subject.

In Governmentality approaches, *problematization* is the process authorities use to embrace populations and define their responsibilities to each other. By articulating what is and what is not a problem or, in this case a barrier, authorities construct a favourable arrangement of people and things. In this respect, barriers are as much about defining the real problems people face, as they are about claiming and ruling in their name. Because advanced liberal governance is often decentralised, this process allows authorities to coordinate their activities without ceding power to each other. Hence authorities rely on translation processes that allow information about their jurisdictions (Rose, 1999).

Building on this approach, I argue *barriers* elide definition because they function as *empty signifiers*. Laclau (2000) defines empty signifier as words or concepts that are meaningful at a high level of abstraction, but which become effectively meaningless when compared across specific or local contexts. An empty signifier indexes concepts that are initially first similar, but through a chaining of concepts becomes so broad as to be effectively meaningless. These concepts e.g. *the people, democracy, freedom, justice* are common in political and especially populist rhetoric (Leclau, 2005; Gasché, 2012).

The existing criticisms of barriers suggest this is a fitting conceptualisation. At a high level of abstraction, barriers are anything that negatively impacts a process. Though there are overlaps, there are no *equivalences* between popular barriers like access, stigma or motivation. Moreover, these concepts themselves break down into chains that include financial cost, the physical environment, prejudice, racism, self-perception and beliefs about self. Without universalities, criticism suggests that barriers are subjective.

Rather than consider ambiguity, multiplicity and a tendency to be subjective weaknesses of barriers, these are the qualities that make them useful. I argue barriers are a discursive tool that allows for the deliberation on how a system ought to be ordered. Liberal governance is perpetually self-critical and thus relies on collecting knowledge, enabling rule from a distance (Rose, 1999; Foucault, 2008). This process *produces* the systems and power relations that knowledge-makers claim to describe. Barriers are not only constructive, but as I will demonstrate, they transform the substance and organization of an area of healthcare knowledge and provision, without any explicit acknowledgement of the transformation.

## Methodology

The sample for this paper includes articles about barriers as well as guidelines, letters and grey literature publications that spoke authoritatively about PrEP barriers in England between 2016 and 2020. I examined 113 texts, produced by seven PrEP stakeholder groups producing discourse about PrEP in England between 2015 and 2020.

Situational analysis uses concurrent and iterative mapping exercises to chart discourse elaboration (Clarke et al., 2018). This article draws primarily from two mapping strategies: social worlds/arenas maps and positional maps which clarify how barriers were deployed within a system of alliances and how the positions taken up in discourse, corresponded to the different social and material needs of those groups.

To determine the social worlds involved in the debate, I looked at the institutional affiliations of the authors of articles, presentations and guidelines on PrEP in England. Institutional affiliation can be used as an index for the researcher’s social world. A researcher’s affiliation reflects access to resources, the nature of their research interest and claims (Hottenrott et al., 2021), which Clarke, Friese, and Washburn, (2018) define as the defining traits of a social world. Based on this and through multiple mapping activities, these seven stakeholder groups were consolidated into three *arenas* based on their shared patterns of concerns and commitments, linking them and their participation in the production of academic research on PrEP barriers and/or the production of guidelines. These arenas also contained networks of people who collaborated to produce discourse about the barriers people faced.

As activists were avid participants in UK PrEP discourse (Young et al., 2021), I paid special attention to activist publications. I refined my sample to UK publications in which activist organizations collaborated with scientific and social scientific actors. By limiting the sample to discourses produced by the most influential activists, this article focuses on *entrepreneurs* most closely involved in PrEP discourse (Clarke, Friese and Washburn, 2018). This information-oriented sampling strategy focuses on most relevant data, rather than a representative sample (see, Flyvbjerg, 2001).

To determine which barriers appear in PrEP discourse, I consulted systematic reviews of PrEP barriers. These reviews (e.g. Coukan et al., 2023, Matacotta et al., 2020, Mayer et al., 2020) identify three key factors: individual barriers, interpersonal barriers, structural^1^ and systemic barriers. Individual barriers tended to include questions about personal motivation, knowledge, side effects and risk perception. Interpersonal barriers primarily dealt with stigma and discrimination. Systematic barriers tended to deal with aspects of PrEP provision that affected accessibility. Based on the barriers identified in these reviews, I considered how each group positioned themselves relative to six barriers: motivation, information, access, side/effects, stigma and risk perception. These positions were translated into the following *positional map* (Table 1).

## Findings

While each network had activist, academic, healthcare and governmental involvement, each network privileged one of these perspectives. Network 1, represented by The National Institute for Health and Care Excellence (NICE) prioritized state interests. This network is mainly composed of epidemiologists and medical experts, with assistance from the Department of Health and Social Care, Public Health England and to a much lesser extent the pharmaceutical company *Gilead Sciences*. Network 2, represented by the British HIV and British Sexual Health Associations (BHIVA/BASSH), prioritized clinical and health system interests. This network is mainly composed of healthcare specialists affiliated with tertiary care institutions. Network 3 is primarily composed of activists, social scientists and critical public health experts. Though there is significant overlap between these three networks, they represent state, healthcare and activist interests respectively.

Each network also had distinct positions on PrEP (Table 2). Network 1’s positioning of the PrEP user as *high risk* groups, represented a more cautious approach, reflective of the formal position of uncertainty about PrEP’s real-world effects taken by NHS England (Nagington and Sandset, 2020), and the moral stance of the conservative government (Mowlabocus, 2019). The category of *high-risk* is dropped in Networks 2 and 3, in favour of clinical and individualistic configurations of the subject. These networks also adopt a more pro-PrEP attitude, with the activist network adåopting the most progressive stance.

## Who is the subject?

If barriers are descriptions of problematic systems that deny entry to a specific kind of person or group, then the subject should remain stable. This is not what happens in English PrEP discourse. Rather, a transition occurs from an initial proposition of PrEP for ‘MSM (men who have sex with men) who are at the highest risk of HIV-infection’, to ‘everyone who needs it.’ This transition illustrates that in the process of debating what is and is not a barrier, a transformation of the subject occurs, alongside major transformations about how HIV-prevention is thought about and organized, and who is considered authoritative.

Early PrEP discourse considered PrEP a minor complement to existing prevention strategies reserved for very high-risk groups. When PrEP was proposed in England in 2015, it was to be provided conservatively to only those at *highest risk* of HIV-infection, approximately, 4500 to 6500 persons, predominantly MSM based on the clinical usage statistics (Foreman et al., n.d). Likewise, the focus on persons assigned male at birth, who have sex with men, is reflected in early recommendations. The NICE determined that PrEP’s “likely place in therapy” would be limited to HIV-negative, adult MSM and trans women as the highest risk communities in England (NICE, 2016). They called for “high impact, appropriately tailored combination prevention strategies and programmes” for MSM and trans women (NICE, 2016). Other groups would be provided PrEP following a comprehensive risk assessment.

Already in these recommendations there is a slippage in the subject. While the NICE do not index the items commonly identified as *barriers* in PrEP literature, they are mentioned. For example, they noted the cost-effectiveness of PrEP is,

…very sensitive to key variables such as HIV incidence, uptake, adherence, trends in condomless sex, and drug costs. The WHO guidelines from 2016 suggest that giving priority for the use of PrEP to the people most at risk of acquiring HIV infection could increase its impact. (NICE, 2016)

Common barriers in the literature like uptake, adherence, trends in condomless sex and drug costs are indexed as *variables*. These are not *barriers, per se*, but factors that influence the cost and impact on the *health service* and which vary by the groups using and behaviours on PrEP. Rather than index uptake, adherence, sexual preferences and the accessibility of PrEP to *barriers*, the NICE index them to variables. This is consistent with their reluctance to approve PrEP. It also aligns with the NHS’ claims that the same variables constituted *uncertainties* that required further analysis (Naggington and Sandset, 2020).

Uptake, adherence, sexual preferences and the accessibility of PrEP were also cast as *issues*, rather than barriers. The NICE also expressed uncertainty about PrEP’s real-world feasibility, writing that, “issues relating to uptake, adherence, sexual behaviour, drug resistance, safety, prioritization for prophylaxis and cost-effectiveness,[as] important to consider, especially at a population level” (NICE, 2016). They were concerned, among other things, that MSM and others at substantial risk were fundamentally unwilling or unable to adhere to PrEP or might increase their risk-taking practices. Concerns about paradoxical raises in HIV rates due to risk-compensation were a common concern at the time. These concerns are cast as issues, not barriers meant to be overcome; issues are vital and unsettled problems, implying insurmountablility.

Limiting PrEP to a small group of MSM and choice heterosexuals deemed high risk was unacceptable to Networks 2 and 3. Front line workers expressed concerns about assessing s*ubstantial* risk and activists were concerned that the proposed policy would limit access. These groups collaborated to produce evidence that even people categorised as low risk are susceptible to HIV, and thus ought to be provided PrEP (Bourne et al., 2017; Young et al., 2014).

As a network with an understanding of the governmental and scientific limitations of PrEP and the needs of patients, Network 2 attempts to strike a more middling position on PrEP. In a section on the cost-effectiveness of PrEP writers for the clinical associations for HIV and sexual health stress that the problem at hand is not the variables, but the way they are managed that influences how PrEP impacts the health system. Firstly, they counter the NICE’s position,

In order to evaluate the cost-effectiveness of interventions it is necessary to use mathematical models …It is important to bear in mind which type of model is used because static models by definition do not capture the full benefit of interventions such as PrEP that prevent new transmissions [as captured by dynamic models] (BASSH/BHIVA, 2018)

Hence, from their point of view the problem of cost is not insurmountable. The more pressing problem by their estimation was the subject. They explain,

… potential challenge that was raised was whether it would be realistic to offer PrEP by risk level, the potential challenge of identifying the target population, and how policy could be implemented selectively to prioritise access to PrEP given the substantial budgetary implications [for the NHS].

While targeting PrEP to the highest risk groups is necessary to make PrEP cost and clinically effective, they note,

It is quite difficult to identify using sufficiently specific clinical criteria for any group of heterosexual people in the UK who would be at sufficient risk. The WHO recommends PrEP for those at substantial risk of HIV acquisition, which is defined as an HIV incidence greater than 3 per 100 person-years. … However, we know that black African women [incidence 0.17] are at substantially more risk of transmission than men… (BHIVA/BASSH, 2018)

The subject appears to be heterosexuals, and MSM at substantial risk. However, clinicians’ ability to appraise *substantial risk* using the WHO recommendations is the real target of their discourse.

In the absence of a clear evidence-base, the guidelines suggest physicians should “make pragmatic decisions with patients about future HIV risk, their need for PrEP and individual-level assessment of the benefit versus potential harms of PrEP” (BHIVA/BASSH, 2018). They justify the partial inclusion of heterosexuals by stressing that heterosexuals tend to be less educated about PrEP, and there “are likely to be differences in cultural beliefs and sociodemographic circumstances that influence adherence and efficacy and further complicate any extrapolation of” the existing clinical studies on heterosexuals (BHIVA/BASSH, 2018). Importantly, cultural beliefs and education *are* barriers for members of Network 3 (see, Witzel et al., 2019) but not for Network 2. For this network, who privileges healthcare perspectives, beliefs and education are both variables and complications. Unlike Network 1 who frames these variables as potentially insurmountable *issues*, Network 2 presents them as variables and *complications*. This aligns with their perspective that in the absence of a clear evidence-base, clinical judgement ought to be prioritised. The subject is also modified. No longer cast as only those at highest risk of infection, this group recommends PrEP to epidemiologically defined groups at the discretion of physicians and supported by clinical evidence.

Network 3 is less ambivalent about barriers than Networks 2 and 1. Pebody and Nutland (2018) use barriers to argue that *individuals*, not clinicians nor the health system, ought to decide PrEP use. In direct opposition to the NICE’s “issues relating to uptake, adherence, sexual behaviour…at a population level” and the BHIVA/BASSH’s claim that physicians should determine who is at risk in light of social and cultural barriers, Pebody and Nutland (2018) strategically redefine candidates as thoughtful, self-aware decision-makers.

Contrary to the other networks, they argue guidelines underestimate “…the overall national picture” which “may hide individuals, groups, communities and networks that are at greater risk of HIV infection” (p.5). They note, people tend to have a *better* idea of their own risk than doctors and write, “People asking for PrEP are likely to have made this choice based on a careful assessment of their personal circumstances, risk and desire for additional HIV prevention” (p.6). Moreover, guidelines tend not to acknowledge “additional benefits… PrEP allows many people to have more enjoyable and satisfying sex, but this isn’t usually included as a factor in PrEP guidelines.” (p.6) In this respect, Network 3 contradicts the position adopted by the other two regarding the factors determining eligibility and risk assessment. Rather than treat cultural differences issues that impact uptake, Network 3 notes individuals are aware of their social context and priorities and thus *know better* than their physician and the government. The problem at hand is due to patients not having *agency* to act on their knowledge and values.

Network 3’s advice hinges on the barriers *patients* face. Pebody and Nutland (2018) note, “International guidelines do not all provide the same advice on starting and stopping PrEP. As some PrEP users may have heard contradictory information, it can be helpful to be aware of what other guidelines say” (p.12). The patient might be well informed, they nonetheless may be *misinformed* by “different scientific interpretations of the limited evidence that is available…[and] *experts* having different approaches to risk and harm reduction” (p.12, my emphasis). This barrier also biases clinicians, “… Should doctors recommend strategies that they are highly confident will be safe, even if they might be difficult for some people to put into practice? Or should they take a pragmatic approach that seeks to reduce risks rather than eliminate them entirely?” (p.12). The language of pragmatic decisions echoes Network 2’s language. However, the autonomy over decision-making is shifted to patients whose motivations may include the additional benefits of PrEP which are considered not clinically relevant by the NICE or BHIVA/BASSH.

Likewise, Peabody and Nutland (2018) strike a more optimistic tone about the cost of PrEP to the NHS. They explain that after 20 years of averted HIV transmissions, most economic scenarios “found that a PrEP programme for MSM in the UK would not only be cost-effective but actually cost-saving.” In this case, the variables and variability of the findings are presented as favouring PrEP use, rather than the more moderate and conservative claims of the other two networks. The link between cost-effectiveness and individual barriers are also more clearly stated. “PrEP is less likely to be cost-effective if it used by people who would probably not acquire HIV anyway or if it is less effective because of low adherence.” (Peabody and Nutland, 2018). This statement gestures toward the barriers *people* face, as opposed to groups. In shifting the focus from MSM, to people who do not perceive or act on their HIV-risk correctly, the NHS would save money. In this instance, Network 3 attempts to foreclose on debates regarding MSM, whilst noting PrEP will be less cost effective among *anyone* who cannot adhere to the protocol for any reason or who may never encounter HIV.

The debates in this section hinge on the factors influencing the effectiveness of PrEP in the UK. This collection of factors is indexed to uncontrollable and unknown *variables, factors*, or *issues* that influence the effectiveness of the intervention. They become modifiable and more certain when termed *barriers*. The risk of transmission becomes more concrete and more closely linked to the individual, who knows better than the policy makers and doctors. Notably, the subject of these statements often slips between doctors, institutions and the patient. Generally, indexing the factors that initially required limiting PrEP to the highest risk groups, the targeted group, transforms from high-risk MSM to the more generalized public. This generalization is reflected in current PrEP guidance.

PrEP should be offered to people, regardless of their gender or sexual orientation who would benefit from a reduction in HIV risk including, people who request PrEP…, people at risk of HIV…people who, regardless of gender or sexual orientation, are likely to have condomless anal or vaginal sex with people at risk of HIV … where HIV risk is likely to be in excess of the background UK population and where benefit outweighs clinical risk of PrEP” (BHIVA/BASSH, 2024).

Though aspects of the previous iterations remain, the subject is changed.

## United Against DIY PrEP

Barriers also operate discursively to constrain the subject. This is evident in the way that barriers are used to differentiate what qualities the idealized user is meant to have, those they lack and those disallowed. In the following example, barriers were deployed strategically to foreclose on DIY PrEP use and consolidate the subject as the *PrEP Candidate*: a person sufficiently empowered to act on their health risks responsibly, but who lack the *specialized* knowledge to use PrEP effectively on their own. All three networks collaborated to install this figure in academic publications through the barriers they argued people face.

Though networks 1 was mostly silent on the subject, networks 2 and 3 had to contend with the problems brought about by individuals accessing PrEP outside the NHS. Prescribing clinicians were in a challenging position balancing their ethical requirement to provide the best available treatment option, given their legal responsibilities to ensure the patient’s safety. Though patients were able to access PrEP through private pharmacies, many of the online pharmacies were considered dubious and clinicians were concerned they could be held liable (McCormack et al., 2016, BHIVA/BASSH, 2018). Without additional funding that normally accompanied commissioning, sexual health centres were poorly equipped to manage the influx of thousands of PrEP users looking for HIV testing and drug monitoring (Girometti et al., 2018). Hence, eliminating DIY PrEP was in the best interest of the health system and clinicians.

Members of Network 3 were also interested in foreclosing on DIY PrEP. While not opposed to DIY PrEP given the role of Iwantprepnow.com and PrEPster in disseminating information about DIY PrEP, the network was cognizant that the government might see this group as justification to limit NHS PrEP provision. Limiting PrEP to DIY PrEP users would neglect marginalized populations, particularly racialized persons and persons living outside London, who relied on the NHS for their HIV information and services. Hence, they collaborated with them to produce discourse that foreclosed on the DIY PrEP user. Through their shared problematizing activities, UK activists and researchers *collaborated* to reframe the PrEP user as the responsible healthcare actor, who can act on their risks independently, but who *also* requires the support of their clinicians and health service to do so safely.

PrEPster used their networks to recruit focus groups and in-depth interviews with early PrEP adopters (Witzel et al., 2018, Witzel et al., 2019, Paparini et al., 2018). They also collaborated with the Terrence Higgins Trust, and clinicians and researchers at University College London to test the authenticity of PrEP sourced outside the NHS (Wang et al., 2019, Wang et al., 2018a, Wang et al., 2018b). Through these articles, the collaborators cast their subject as a responsible and able patient who could overcome common barrier, but one who lacks the specialized knowledge of their bodies’ inner workings to use PrEP safely, without physician oversight.

Both groups also identify the barriers early adopters faced in accessing and paying for PrEP. This included “frustrations with the clinical support services offered, citing lack of consistent care or information, and health workers who were not always aware of the tests and procedures necessary for PrEP use” (Paparini et al., 2018). They illustrated how trustworthy information about drug monitoring and side effects was necessary: fear of kidney damage led *DIY PrEP users* to discontinue PrEP.

Despite this, Paparini and colleagues (2018) DIY PrEP described users as, “highly motivated … keen and able to experiment with new strategies for HIV prevention, even in the context of partial information and the need for significant personal motivation” (p.12). Their sample were also more educated and older than the conventional sample of PrEP users. They “clearly expressed that they would have preferred a PrEP service in their sexual health clinic to begin with”. Accordingly, researchers determined that the DIY PrEP user is considered *not* to face barriers of motivation or education but lacking access due to the dearth of insurance coverage for PrEP. Either they were unable to afford the premium or could not access PrEP through that route, because it was not widely available. In this respect, these subjects are framed as exceptional to the normal healthcare actor.

However, Paparini et al. (2018) note taking PrEP without support, “carries a number of risks, not least of reduced effectiveness of PrEP against HIV” making it crucial that PrEP users receive as much support and information as possible. Recent studies have confirmed the overall safety of *interPrEP* formulations, “PrEP users need confirmation of the authenticity of their prescription…if new sellers or manufacturer enter the market, it will be important to reconsider safety issues.” (p.13) This need can be met through resource reallocation and training for the providers, rather than the patient: “As PrEP use is scaled up, healthcare workers need to be supported in developing PrEP-specific skills to address the needs of PrEP users.” Patients “benefit from clear and standardised guidance on the clinical monitoring needed to assess long-term drug effects, and support around drug-drug interactions.” (p.13)

A partnership between the THT, PrEPster and researchers at University College London (Wang et al., 2018, 2019) could work together to support the figure who could act independently of health system supports, but who lacked the specialized knowledge to act entirely on their own. They collaborated to determine whether generic PrEP from online pharmacies was safe to use. While the PrEP pills they tested were safe, their findings underscored that DIY PrEP is needlessly dangerous, since sourcing PrEP online,

…circumvents established safeguards against aspects of quality control, from manufacturing to distribution…As for all generic drugs, without specialised analytical equipment, it is impossible to inform individuals who purchase generic PrEP online of the quality or authenticity of the drugs. (Wang et al., 2019, p. 2)

Conclusively, while early adopters are *motivated* and *informed* about how to prevent HIV and are able to *adhere* to the protocol effectively—provided they are members of a supportive community (Paparini, et al. 2018)— they can never be *fully* empowered to manage their health risks on their own. They lack the specialized knowledge and equipment necessary to take PrEP safely.

Again, we can see how barriers are deployed strategically to designate how a person ought to know, and act with their bodies. Barriers can help distinguish the knowledge a person ought to have about themselves from knowledge they do not require. Barriers also foreclosed on inconvenient practices, even at the expense of patient empowerment, when that empowerment clashed with existing health services. Consequently, the subject of PrEP discourse takes on a bit of a liminal quality. The privileged advanced liberal subject tends to be cast as autonomous, enterprising but prudent. This is not strictly how the PrEP user is cast. Ideally, they are autonomous, but full autonomy is impossible.

## Discussion

Despite the scientific evidence, PrEP conflicted with the style of thought underpinning HIV-services in England. It existed radically outside the system of relations that made up HIV-prevention in England. Not only was it politically unfavorable, as others have observed (Dodds, 2021, Nagington and Sandset, 2020, Mowlabocus, 2019) but it proved to be *impossible* to implement within those current relations. The changes necessary to make PrEP possible within the NHS was not only achieved through policy change, advocacy and scientific research, but by stating the problems the target community faced. Notably, in differentiating which problems were surmountable barriers and insurmountable problems, authorities tended to implicate themselves.

To make commissioning the best available solution, a new subject position is created with novel relationships to pastoral and medical knowledge. Barriers are strategically important in this context, as a mode of revisualizing the existing set of relations and redeploying them to encompass the unintelligible. Decades of HIV denialism had rendered the notion that anyone can contract HIV a reluctant utterance (Epstein, 1996). Yet, by way of a series of negotiations over the barriers each of the risk groups experienced, the subject is homogenized. Important to note here is that what were previously antagonistic needs or problems become solvable by way of *changing the subject*. In this sense, barriers are not strictly *representations* of the problems within a system for a given group, constructive of their inherent values. By indexing all problematic aspects of the system, an empty signifier becomes representative of the system as a whole, and thus allows us to visualize what is beyond the system of thought in which that discourse operates (Gasché, 2012, Laclau, 2000). This is what we discover: PrEP is not *for* MSM or risk management, but for the sense of security all people desire whilst having sex. To resolve the barriers initially faced by those at risk, nominally gay and bisexual men, that subject is erased. This shift eliminates the anatagonisms Race (2016) suggests made PrEP a *reluctant object*—without acknowledging the moral or political aspects of that reluctance. The unthinkable idea, that *everyone* is at risk and thus requires PrEP is rendered a matter of fact.

Rose (1999) has argued that for liberal governance to proceed, translation needs to occur between its disparate elements. Barriers allow for these translations to occur by being just intelligible enough to isolate a problem, but vague enough for that problem to be characterized differently. The networks debating PrEP provision used these shared concepts to collaborate on a shared problem without conceding authority or their own position on that problem. This fragile peace allows for certain kinds of activism to occur within medicine. A somewhat flaccid version of the biological citizen that PrEP could have fostered results: a person who is engendered to act on themselves but not to *think* with biomedical interventions. Users are expected to have knowledge of their sexual desires and needs, and the risks associated with them, but are not expected to possess the deep somatic knowledge of what occurs at the level of drug interactions, organs and cells. This person who is expected to act on their bodies at the molecular level, but whose access to that knowledge is circumscribed, is curious and adds yet another ambivalence to the PrEP user subject position (Gaspar et al., 2022, Sandset, 2023).

If there are theoretically infinite possible variations of healthcare provision, an important question for critical health scholarship is an examination of the ways in which this variation came to be and whether this is the best possible iteration. As illustrated above, PrEP provision could have taken multiple forms, some less desirable than others. Though DIY PrEP may not be desirable, it offered, for a moment, a possibility for PrEP users to know their bodies in an entirely new way: a possibility that they be trained to see and know the chemicals and processes that act in their bodies and learn to see and act on themselves in an entirely new way. From here one might envision a new form of biomedical subjectivity, an art of the self and aesthetic of the body at the molecular level. The fact that this possibility was actively foreclosed upon requires further consideration.

## Conclusion

This case study provides a theoretical and methodological basis for a critical analysis of barriers discourse in healthcare. Barriers are a *strategic* description of the problems patients face, that facilitate the alignment and contestation of related descriptions. These descriptions facilitate the *construction* of a preferential arrangement of elements based on the desires and knowledge of groups representing aspects of healthcare. Barriers are thus not simply factors that explain non-compliance, but are a strategy of governance that warrants future research.

This article underscores the value of a governmentality approach for understanding how barriers are used in discourse. Rather than assume they are representations or strict constructs, this approach allows us to see how barriers translate the multiple representations of the problems and desires of authorities in healthcare. The urgent problem created by PrEP in English HIV-provision required its elements to struggle against each other to sustain themselves and collaborate to coral their subjects. By discussing barriers, variables and issues these authorities structure healthcare ‘at a distance’, without explicitly appearing to exert political will. The case study presented illustrates how examining what counts as a barrier reflects the values and desires of groups vying for authority over the subject. Further, it allows us to see how in translating, they not only exerted their will over a static population but in effect transformed the processes of HIV-care and subject of HIV-discourses radically.

## Figures and Tables

**Table 1:** Positional Map of PrEP Barriers

	**Favorable**	Motivation is not a concern	Patients have a better understanding of their health needs than the clinician	Expanding access to PrEP is paramount	Side effects are mostly not a concern.	Stigma is a major problem	Patients have a better understanding of their risk than clinicians
**Position**	**Moderate**	Only MSM and select members of risk groups are motivated	PrEP users tend to be more informed than MSM generally	Access must be accompanied by resources	Lack specialized knowledge to monitor PrEP’s effects	PrEP users tend to be more resilient than the general population	Risk is poorly defined in current guidelines
	**Against**	Motivation is a real concern among prospective PrEP users	Misinformation is a real concern among prospective PrEP users	PrEP is not approved and therefore not a barrier. However, cost is a concern.	Side effects will lead to discontinuation	[Missing]	Risk compensation is a real concern among prospective PrEP users
		**Motivation**	**Information**	**Access**	**Effects**	**Stigma**	**Risk**
	**Barrier**						

**Table 2:** Position on PrEP, Candidate and Orientation by Network

Position		Group	
	State (Network 1)	Healthcare (Network 2)	Activist (Network 3)
**Candidate**	High Risk Groups (MSM)	Epidemiological Groups (MSM)	Anyone who needs it (PrEP Candidates)
**PrEP** **Orientation**	Daily Pill Only Cautious	Daily Pill or Event-based. Moderate	Daily Pill or Event-based Favourable

## Footnotes

1 It is often unclear how studies differentiate between interpersonal and structural barriers. Here structural barriers refer to any problems that are related to the organization of the health system, its policies, governance or institutionalised practices.
